# Advancing spatial repellents for malaria control: effectiveness and cost-effectiveness of a spatial repellent under operational use in Northern Uganda—study protocol for a cluster randomized controlled trial

**DOI:** 10.1186/s13063-024-08378-1

**Published:** 2024-08-22

**Authors:** Esther Nakyaze, Suzanne Van Hulle, John Hembling, Emmanuel Arinaitwe, Momar Mbodji, Mary Grace Alwano, Felly Christine Lamwaka, Stephen Tukwasibwe, Samuel Gonahasa, Fang Liu, John P. Grieco, Nicole L. Achee

**Affiliations:** 1 Catholic Relief Services, Kampala, Uganda; 2 Catholic Relief Services, HQ, Baltimore, MD USA; 3 Infectious Diseases Research Collaboration, Kampala, Uganda; 4 University of Notre Dame, Notre Dame, IN USA

**Keywords:** Malaria, Spatial repellent, Transfluthrin, Vector-borne diseases, Mosquito vectors, Incidence

## Abstract

**Background:**

Spatial repellents (SRs) have been widely used for the prevention of mosquito bites, and preliminary findings suggest efficacy against both malaria (1) and *Aedes*-borne viruses (2) but their effectiveness in reducing mosquito-borne diseases under operational use has never been evaluated. SRs have the potential of being critical tools in the prevention of mosquito-borne diseases in contexts where typical vector control strategies, such as insecticide-treated nets (ITNs) and indoor residual spraying, are inaccessible or underutilized such as among displaced persons or in emergency relief settings.

**Methods:**

Children will be enrolled in 3 separate cohorts to establish the effectiveness of SRs in reducing malaria infection in different distribution channels. One cohort will estimate the direct effect of the SR distributed through a reference channel (study personnel distribution). The two remaining cohorts will estimate the protection of the SR distributed through a voucher channel and the Village Health Team channel. Cohorts will be followed twice a month (approximately every 15 days): during the first scheduled household visit in the month, a blood sample will be taken for malaria rapid diagnostic test (Monthly Visit #1); and, during the second scheduled household visit, a blood sample will only be taken if the participant has a recent history of fever (Monthly Visit #2). The incidence of malaria in each cohort will be estimated and compared to the reference cohort to determine the benefit of using a SR in an area with high, year-round transmission of malaria.

**Discussion:**

This study will address the knowledge gap of whether or not SRs are effective in reducing human malaria disease in humanitarian assistance and emergency response settings in sub-Saharan Africa where underlying transmission rates are historically high and ITNs may or may not be widely deployed. This research will inform policy makers on whether to recommend SRs as a means to further reduce malaria transmission for such operational programs.

**Trial registration:**

ClinicalTrials.gov NCT06122142. Registered on November 8, 2023.

## Introduction

### Background and rationale {6a}

Spatial repellents (SRs) are a promising new vector control paradigm that could add to the existing armamentarium for malaria prevention [1, 2]. SRs such as mosquito coils have been shown to reduce mosquito biting [3, 4]and, in studies conducted in Indonesia [5, 6] and China [7], to reduce malaria transmission in human populations. Recently, SRs have also been proven to reduce *Aede*s-borne viruses [8]. Although the value of SR is applicable across a wide range of scenarios, evidence is required to demonstrate the effectiveness of SRs under operational use conditions. One scenario in which SRs have potential for significant impact is in humanitarian relief efforts. People who have been displaced due to conflict or environmental disasters very often arrive in an exhausted, weakened, traumatized state, already suffering from or vulnerable to infectious diseases and malnutrition. They may lack access to health services and health commodities such as insecticide-treated nets (ITNs), and temporary structures to accommodate them are often substandard or semi-enclosed, providing little protection from mosquitoes.

As of 31 March 2023, the United Nations High Commissioner for Refugees has registered 1,532,168 refugees in Uganda, of which 867,391 are from South Sudan and 487,044 are from the Democratic Republic of Congo. Uganda has a unique refugee policy, whereby refugees are given a 30 × 30 plot to construct their structures and farm on this land. Refugees are placed into different areas based on their date of arrival. Refugees live among host communities in the same area and have equal access to health facilities and other services.

Malaria is highly endemic throughout all of Uganda and accounts for 31.1% (14,381,183/46,261,118) of all reported outpatient visits and 25% (844,965/3,385,664) of all admissions, placing a heavy burden on the health care system [9]. This is especially true in the north of the country, where fighting in South Sudan has displaced tens of thousands of South Sudanese people who have been forced to seek refuge in northern Uganda. The prevalence of malaria according to rapid diagnostic test (RDT) in all refugee settlements across the nine hosting districts was 36.6% for children 6–59 months [10]. Refugees live in grass thatched roof houses or in tarpaulin structures, and at times in areas of rapid over-growth that provide ample breeding space for mosquitoes.

Along with many other multilateral and international organizations, Catholic Relief Services (CRS) provides humanitarian assistance to victims of natural and man-made emergencies across the world, including three longstanding conflicts in Uganda. Because of the high burden of malaria and the history of CRS’ efforts in this region, the site was selected to evaluate the effectiveness of a new SR product formulated to last up to 1 month for the prevention of malaria using a cluster randomized control trial (cRCT) study design. The study will enroll children 6 months to 59 months of age as study participants into three separate cohorts and followed to determine the time to first malaria infection with monthly RDTs. The study will include separate cohorts to measure the direct effect of SRs as well as the protection to participants using varied distribution channels: paid study personnel (reference arm-positive control), Village Health Teams (VHTs), and a voucher system. The voucher system will be modeled based on CRS’ past experience using vouchers in bed net distributions and emergency response programs.

This evaluation will serve as an effectiveness trial of SR products for sub-Saharan Africa emergency response and recovery contexts. Evidence will inform health impact and equivalence of impact through different ‘real-life’ delivery systems for donor market introduction. The cRCT will evaluate operational effectiveness against malaria infections in a rural African environment linked to displaced persons where ITNs and indoor residual spraying (IRS) may be constrained. Findings and operational use guidance will be submitted to the World Health Organization (WHO), in coordination with the Ministry of Health (MOH) in Uganda.

Despite the scale-up of effective tools for the prevention and control of malaria, this disease remains one of the primary causes of morbidity and mortality in sub-Saharan Africa. New tools are needed to address the threat of insecticide resistance and outdoor biting vectors and to sustain the drive to elimination. SRs such as mosquito coils have been shown to reduce mosquito biting [3, 4] and, in studies conducted in Indonesia [6] and China [7], to reduce malaria transmission in human populations. Recently, SRs have also been proven to reduce *Aede*s-borne viruses [8]. The WHO has developed methods to evaluate the efficacy of new SR products; however, the effectiveness of an SR intervention delivered under programmatic conditions has not been evaluated. It is unlikely that efficacy estimates derived from tightly controlled phase III trials will be realized in program settings. This study will address the knowledge gap of whether or not SRs are effective in reducing human malaria disease in humanitarian assistance and emergency response settings in sub-Saharan Africa where underlying transmission rates are historically high and the use of ITNs may be challenged. This research will inform policy makers on whether to recommend SRs as a means to further reduce malaria transmission for such operational programs.

### Objectives {7}

The primary objective of the study is to demonstrate and quantify the effectiveness of a SR product, in reducing malaria infection in human cohorts under operational program conditions. The design will be a prospective cRCT representing an operational research study.

To compare the effectiveness of SR against malaria infection (both first-time and recurrent) between each of the two operational program delivery mechanisms (1. VHTs delivering SR directly to participants (VHT); 2. participants redeeming a voucher for SR at a central location and applying it in structures themselves (Voucher)) vs a reference distribution channel (positive control; study team personnel delivering product directly to participants at their homes).

Secondary objectives are (1) to estimate the cost-effectiveness of SR distribution by each distribution channel (VHT and Voucher) compared to the reference distribution channel, and (2) assess the safety of the SR product.

### Trial design {8}

The study design will be an exploratory, parallel-group, three-arm, cluster randomized controlled trial. Children aged ≥ 6 months to ≤ 59 months will be enrolled in 3 separate cohorts (16 clusters per cohort) to establish the effectiveness of different distribution channels; one cohort to estimate the direct effect of the SR using a reference channel (study personnel distribution); one cohort to estimate the protection to persons using a voucher to receive the SR; and one cohort to estimate the effect of the SR distributed by VHTs. Cohorts will be followed and RDT will be conducted at least once every 4 weeks to test for malaria or whenever a participant reports a recent history of fever.

## Methods: participants, interventions, and outcomes

### Study setting {9}

Bidibidi Refugee Settlement located in Yumbe District, Northern Uganda, is currently the second largest refugee settlement in the world. About 6% of the population in Bidibidi is living in grass thatched or Aluzinc roof houses with over 20% living in tarpaulin structures, with the majority of those being women and children under 18. Residents access medical care (health facilities, clinics, hospitals, Community Health Worker programs) by walking or cycling. Most health facilities are nearby and at most within 5 km of their home. Refugees have settled in the Bidibidi Refugee Settlement according to 5 Zones, with first arrivals going to Zone 1, then to Zone 2, similarly to other Zones. All factors pertaining to population movement have been taken into account in the loss to follow-up rate.

There is stable, perennial malaria transmission in 95% of the country, with *Anopheles gambiae *s.l. and *An. funestus *s.l., the most common malaria vectors. *Plasmodium falciparum* accounts for 98% of infections; both *P. vivax* and *P. ovale* are rare and do not exceed 2% of malaria cases in the country [11]. Uganda’s climate is largely tropical with two rainy seasons per year, March to June and October to November. The northern region, which forms one quarter of the country lies outside the tropical belt, and hence experiences only one rainy season, March to October [11].

This research study will be focused in Bidibidi Refugee Settlement Zones 1 to 4, as the study population is more stable than in other Zones. The 48 clusters to be delineated for subject enrolment and follow-up will include 70% refugee settlements and 30% adjoining host-community villages.

### Eligibility criteria {10}

All children aged ≥ 6 months to ≤ 59 months who report sleeping in selected study clusters > 90% of the nights of each month will be eligible for inclusion in the study. Children < 6 months to > 59 months with Hb ≤ 7 g/dL with no other serious illness will also be excluded from the study.

Persons who are participating in another clinical trial investigating a drug, vaccine, medical device, or procedure will also be excluded from the study. The written consent of the parent or legal guardian of each child will be required for inclusion in the study.

Inclusion/exclusion criteria are in Table 1 below (all inclusion criteria must be met to participate in the study):

**Table 1:** Inclusion and exclusion study criteria

Inclusion criteria	Exclusion criteria
Children ≥ 6 months to ≤ 59 months	Children < 6 months and > 59 months
Children ≥ 6 months to ≤ 59 months with Hb > 7 g/dL and no other serious illness	Children ≥ 6 months to ≤ 59 months with Hb ≤ 7 g/dL with signs of other serious illness or Hb < 7 g/dL with signs of clinical decompensation
Sleeps in cluster (i.e. study area) ≥ 90% of nights during any given month	Sleeps in cluster (i.e. study area) < 90% of nights during any given month
Not participating in another clinical trial investigating a vaccine, drug, medical device, or a medical procedure during the trial	Participating or planned participation in another clinical trial investigating a vaccine, drug, medical device, or a medical procedure during the trial
Provision of informed consent form (ICF) signed by the parent(s) or guardian	No provision of ICF signed by the parent(s) or guardian

### Who will take informed consent? {26a}

The study will be explained and consent form read in the local language spoken in the settlement of the following populations:

- All heads of household (HH) in the selected 48 clusters during the mapping and census activities.
- Parents or guardians in the 45 HHs randomly selected in each cluster to hang SR in the HH.
- Parents or guardians of eligible children enrolling into the study in the 45 HHs randomly selected in the 48 clusters.

The study staff will explain the research in the local languages, allow time for answering questions. Once all concerns are addressed, ICFs written in the local language will be signed or a thumb print taken (either on paper or electronically). It will be stressed that entry into the study is voluntary and they may withdraw from the study at any time for any reason without any penalty. If a participant withdraws, their data will remain confidential and used in analysis up until the date of withdrawal. Consented participants will be assigned a participant identity code (or ID card) on enrollment. Consented participants will be screened for inclusion/exclusion criteria.

All individuals from whom written consent is obtained and who cannot sign their names will provide a thumb print on the consent form for documentation to signal their understanding and willingness to participate. A witness not associated with the study will sign the consent form indicating that the ICF was read, and that participation and thumb-printing were given willingly without coercion. Time will be granted to those individuals who would wish to make consultations with their family members before signing.

For participants who cannot read or write, the following procedures will be conducted:

- The staff member will ensure whenever a participant who cannot read or write is consented, a literate impartial witness (a family or community member of the parent/guardians’ choice) who is not affiliated with the study will be identified and present during the informed consent process.
  - ◦ The witness must be present during the entire consent process.
  - ◦ The language of ICF used should be the one best understood by both the potential participant or guardian/Legally Authorized Representative (LAR) and the witness.
- The study staff member will read the ICF, pausing frequently to allow the potential participant, guardian/LAR, or witness to ask questions.
- The witness will be asked to verify to the best of his/her knowledge that the potential participant understood and agreed with the information provided during the consenting process and all the questions or concerns were addressed satisfactorily.
- The potential participant or guardian/LAR will be directed by the staff on how to put his/her thumbprint on the appropriate box (both copies). The witness will be verbally instructed to write the name and date for the participant. The witness will then be instructed to write his/her name, sign and date the consent forms in the appropriate spaces provided for the witness.

As part of a cluster-wide mapping of HHs, informed verbal consent will be obtained from all heads of HH in the 48 clusters. All heads of HHs will be approached and read the ICF. The study Data Enumerators will confirm if the head of HHs understands the document and, if eligible, that there is a possibility that they might be selected to receive and hang SR for the duration of the study.

After completing the cluster-wide mapping and HH member listing, 45 HHs will be randomly selected from each of the 48 clusters and informed written consent will be obtained to receive SRs. All parents/guardians of these 45 HHs will be approached and read the ICF. Before being asked to sign the ICF, the study enumerators will confirm that the parent/guardian understands the document and that the SR product will be placed in their homes by either study personnel, VHTs, or they need to redeem the SR with a voucher every month. The ICF will also ask that they report to the nearest health facility and/or call the study program manager if they experience any adverse events (AEs) or serious adverse events (SAEs) of interest. AEs and SAEs of interest will be described verbally and will be provided in written form.

Among the 45 selected HHs, a cohort of children will be enrolled to estimate the impact of the SR product on the incidence of malaria disease in the three study arms. In HHs with multiple children that meet the study inclusion criteria, all eligible children will be invited to participate; however, only one will be randomly selected to be enrolled in the study, if eligible. Parents/guardians of children selected to participate in the cohort will be asked to provide consent. An attempt to obtain written, informed consent from both parents of the child will be made, but consent from only one parent will be required for participation (national protocols will be observed).

If parents/guardians of participants consent to enroll in the study, the study team will screen eligible children. After screening children for eligibility, the children will be enrolled. Eligible children will then be tested and treated for malaria free of charge during the study.

### Additional consent provisions for collection and use of participant data and biological specimens {26b}

Not applicable—there is no anticipated future use of participant blood samples. These samples will be taken only for purposes of RDT diagnosis of malaria infection in the current study.

## Interventions

### Explanation for the choice of comparators {6b}

The study will include separate cohorts to measure the direct effect of SRs as well as the protection to participants using varied distribution channels: paid study personnel (reference arm, positive control), VHTs, and a voucher system.

#### Voucher distribution channel

Trained study staff will confirm how many SR products are needed per structure. The voucher will be a small plastic card with a QR and/or barcode that is programmed with the total number of SR products that will be provided monthly to each head of the HH. The head of HH will retain the voucher.

For the initial month of the study, CRS staff will distribute the voucher and provide the SR during a HH visit along with training on product placement and handling. HH heads will be instructed where (central distribution location) and when (every month) they can redeem the SR product(s). For the remaining 11 months, HH heads will redeem their SRs. Vouchers will be redeemed at central distribution locations in the cluster, chosen by the voucher clusters’ community, for example, a health clinic, or food distribution point. Reminders to redeem their SR will be put in place for this distribution channel.

At the central distribution location, the distributor (trained as part of this study) will scan the voucher QR and/or barcode with an electronic device (e.g., tablet or smartphone). By scanning the voucher, the distributor will know and provide the exact number of SR product(s) to distribute to the HH head. The trained distributor will also request that heads of HHs return their old SR product to the central distribution location (for safe disposal) in exchange for the new ones. If there is a discrepancy in the number of returned SR products versus new ones to be issued, the distributors will still issue the total assigned number of new SRs. They will record reasons for the discrepancy. The CRS study Program Manager will then follow-up on these discrepancies to ensure safe product disposal according to the manufacturer’s instructions. If a HH size changes, the HH head will inform the central distribution location or CRS study personnel, which will kick-off a process by which CRS staff will check the new HH dimensions and re-issue a new QR code on a new voucher corresponding to the updated number of SRs to receive.

#### VHT distribution channel

Uganda’s national community health worker program is organized as VHTs who serve as a HH’s first point of contact for various health-related issues. Working primarily as volunteers, VHTs are ordinary community members who receive basic health care training to provide home visits and health management services. CRS will recruit approximately 32 VHTs to distribute SR, collect and dispose of old SR, and provide HH-level education about SRs. VHTs will be recruited from the existing VHT pool, which was identified and trained by the MOH. Lists of active VHTs are available at the health facilities and with the health cluster lead agency. All the VHTs in the refugee settlement have experience implementing integrated Community Case Management (iCCM) for childhood illness and the provision of other interventions such as ITN, immunization, and health education. Once recruited, the VHTs will receive additional training with regards to study procedures around the SR product itself, how to hang, remove, and safely dispose of it, as well as documenting and monitoring the process. VHTs recruited for study activities will also receive a remuneration for their activities based on locally approved rates.

Upon enrollment into the study, HH participating in clusters assigned to the VHT distribution channel will be visited monthly by a trained VHT. During the home visit, the VHT will distribute the appropriate number of SRs based on measurements for all structures in the HH. They will also retrieve used SR and dispose appropriately. The VHTs will track distribution and retrieval and follow-up on discrepancies as needed to ensure safe product disposal according to the manufacturer’s instructions. During the home visits, VHTs will share key educational information about malaria prevention, the SR, including appropriate placement on the walls, safety, side effects, etc. The study team will check to see if the SRs are hung appropriately and address any issues with the residents. VHTs will address any questions or concerns the participating HH might have.

#### Study personnel distribution channel (reference arm)

In the reference arm, the SR products will be delivered and replaced by paid study personnel throughout the intervention period. The study personnel will distribute the SR every month during a data collection home visit. They will also retrieve used SR to ensure proper disposal of SR. Study personnel will track distribution and retrieval and follow-up on any discrepancies as needed to ensure safe product disposal.

CRS study staff will be employed to ensure proper storage of unused products at site and supply management and coordination for the timely replacement of products in the reference arm. The CRS study team will also perform periodic, unannounced spot checks in a random 10% of enrolled HHs during intervention to monitor SR product compliance (installation according to manufacturer specifications) and ITN usage, if available.

### Intervention description {11a}

The SR product used for the study will be a new formulation of transfluthrin, Mosquito Shield™, a passive emanator that releases active ingredient (AI) over a period of up to 4 weeks. SRs, like the Mosquito Shield™, are devices containing volatile chemicals that disperse in the air under ambient conditions (no requirement of electricity or heat to volatilize); they can be placed inside or around houses. The volatile chemicals introduced into the air repel mosquitoes from entering the treated space and/or disrupt human biting and feeding habits, possibly impacting their survival and reproductive behavior [12, 13]. SR products are envisioned to complement and enhance existing vector control methods due to the continual release of volatile AI which precludes the requirement of mosquito contact with a treated surface (i.e., ITN, IRS) providing: (1) protection against daytime, early-evening biting; (2) protection in enclosed/semi-enclosed and peri-domestic spaces; (3) a range of formulation options to fit context-specific application requirements thereby facilitating health systems strengthening; and (4) increase coverage of vector control over traditional methods. In addition, SR product AIs have demonstrated increased attraction to oviposition cues that could intervene in the vector life-cycle or enhance combination interventions (i.e., push–pull) that other interventions do not reach and have demonstrated an effect against insecticide-resistant vector species linked to malaria transmission [14, 15].

Transfluthrin is widely used in mosquito coils and other HH pest control products worldwide. The emanator consists of a pre-treated medium with a standard amount of transfluthrin that will be present throughout the treated space continuously based on a standardized 4-week replacement schedule. Products will be positioned along interior walls (approximately 2–3 m above ground) according to manufacturer specifications of 2 units per 9 m^2^. More than one emanator may be applied in a HH depending on the size of the house. The product will have a unique code associated with an individual distribution channel, which will be recorded at the time of installation and replacement.

### Criteria for discontinuing or modifying allocated interventions {11b}

If a study participant chooses to end their study participation, the study staff will respect the individual’s decision without penalty. Participants who withdraw from the study will still be eligible to receive care from government health facilities. If a study participant no longer meets the study’s inclusion criteria and/or based on AE and/or SAE clinical assessment, staff may terminate participant participation at any time during the study.

### Strategies to improve adherence to interventions {11c}

In order to promote adherence to intervention protocols, standard operating procedures will be developed for clinical tests, drug administration, distribution modalities, and all other study processes. Study staff will be employed and trained to ensure the timely replacement and accurate placement of the SR products. Additionally, they will perform periodic, unannounced spot checks to confirm the SR product is properly installed. If study staff observe a product has been moved after application during a scheduled product replacement, the move will be recorded for use in HH compliance assessment. If necessary, study staff will re-engage with heads of HHs on the importance of maintaining original product placement. Overall product coverage will be estimated based on total HHs recorded having product volume at time of replacement according to manufacturer specifications (2 units/9 m^2^).

### Relevant concomitant care permitted or prohibited during the trial {11d}

While the standard-of-care for clinical management of malaria and vector control interventions (e.g., ITNs, IRS) will not be withheld in any of the distribution channels, these interventions will be monitored and recorded throughout the study. ITNs continue to be distributed through mass campaigns every 2 years with the latest distribution conducted in early December 2023 in Yumbe District, where the Bidibidi Refugee Settlement is located. ITNs are also distributed regularly to pregnant women during antenatal and postnatal care services at health facilities in the Bidibidi Refugee Settlement, and to children under five during expanded immunization programs. IRS started in December 2022 and is expected to occur annually, with the latest conducted in December 2023. At enrollment, eligible children will be provided a treatment dose of artemether-lumefantrine (AL) free of charge for malaria parasite clearance and a new ITN. In addition, study participants will be provided treatment for malaria infection throughout the follow-up period. Participants will be encouraged to continue ITN use and not instructed to avoid alternative vector control tools (e.g., coils, topicals, aerosol sprays, repellents) which will allow for an estimation of the SR effect assuming all other measures are still occurring for malaria prevention, essentially providing insight on an additive benefit above that provided by currently recommended WHO malaria preventive measures.

### Provisions for post-trial care {30}

Not applicable—the study will not provide post-trial care.

### Outcomes {12}

The primary outcome measure of this study will be the effectiveness of SR as measured by RDT confirmed malaria infection incidence (both first-time and recurrent), following the deployment of SR, between each of the two operational program delivery mechanisms (VHT and Voucher) versus the reference distribution channel.

Secondary outcome measures include:

1. Cost-effectiveness of SR distribution by each distribution channel (VHT and Voucher) compared to the reference distribution channel.
2. AEs and SAEs as measured by solicited and unsolicited reports during the intervention period (12 months). The mean, minimum, and maximum frequency and percentage of AEs and SAEs across clusters among enrolled participants will be summarized by distribution channels.

### Participant timeline {13}

**Table:** 

	**Study period**						
Timepoint	**Q3 2023**	**Q4 2023**	**Q1 2024**	**Q2 2024**	**Q3 2024**	**Q4 2024**	**Q1 2025**
Ramp up phase							
Census and mapping		**X**	**X**				
Intervention							
Allocation			**X**				
Informed consent			**X**				
Screening			**X**				
Follow-up				**X**	**X**	**X**	**X**
Assessment							
Final analysis							**X**

### Sample size {14}

**Fig. 1:**
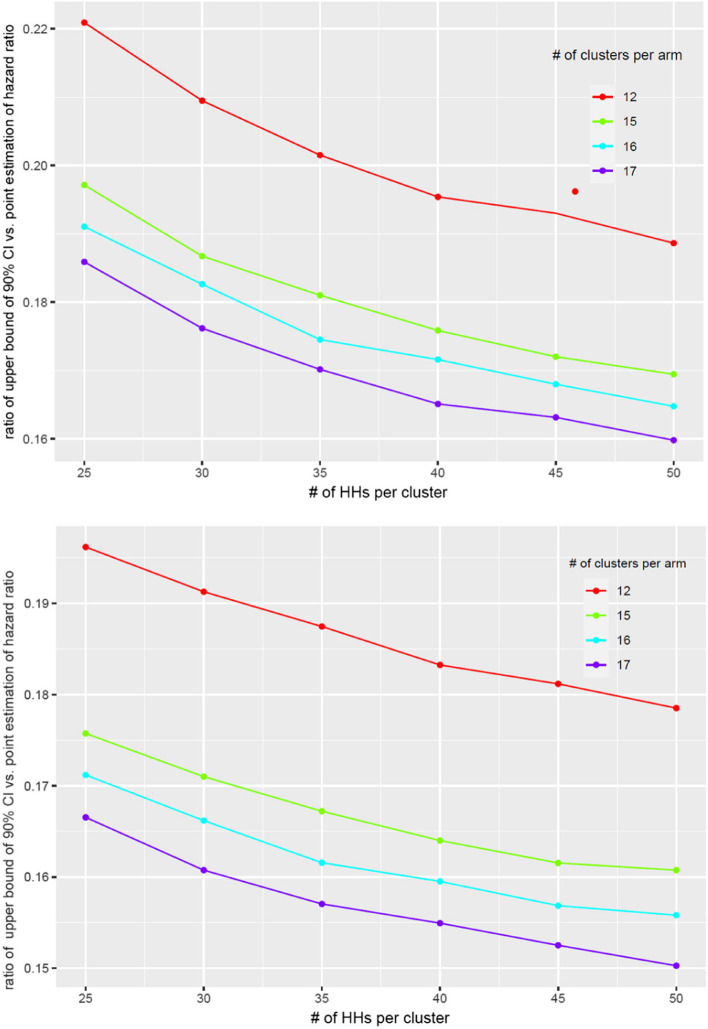
The precision around the intervention comparison at different numbers of clusters and HHs per cluster

The sample size of the number of clusters and the number of HHs per cluster is based on the assumption of a 40% between-cluster coefficient of variance; a 12-month intervention period; and a 10% loss to follow-up. Since the primary objective is to quantify the difference in effectiveness between each of the two real-life distribution channels and the reference without formal hypothesis testing, we focus on determining a practically feasible sample size that yields a satisfactory precision for the intervention comparisons on the malaria incidence rate. The previous data suggests that the baseline incidence rate in the study area is around 0.5 to 1 per person-year. Figure 1 provides the precision around the intervention comparison at different numbers of clusters and HHs per cluster. When the number of clusters is 16 per arm and the number of HHs per cluster is 40, the upper bound of the CI for the hazard rate (HR) between a real-life delivery system vs. the reference ranges from 1.30 to 1.33 for baseline HR of 0.5 to 1.0 per person year, and the corresponding lower bound ranges from 0.77 to 0.75 if the estimated HR is 1. Further increasing the number of clusters or the number of HHs per cluster does not significantly shorten the CI width and increase the precision of the HR estimation. Given the precision analysis for different sample sizes, we will recruit 16 clusters with 45 HHs per cluster for each of the 3 intervention arms; altogether, 2160 HHs will be recruited.

### Recruitment {15}

Prior to recruitment mapping will be conducted to obtain demographic details of HH members in each of the 48 clusters. Information regarding the study will be provided during the stakeholder and community leaders’ inception meetings through study personnel and VHTs, targeting parent(s)/ guardian(s) of potential participants in the community, community leaders, and organizations working with refugees and host communities. Information will be provided through community meetings held at local venues, brochures posters at health facilities and community meeting venues with study staff contact information (in English, Juba Arabic, Kakwa, Lugwara-Ti), study staff attendance at sponsored opinion and religious leaders’ meetings, women groups and men groups, and engagement with tribal and government leaders in the settlement area.

## Assignment of interventions: allocation

### Sequence generation {16a}

The unit of randomization for the SR intervention delivery channel will be a cluster. The study statistician will have access to malaria prevalence and census data that will be collected during the ramp-up phase of the study and may be used to inform potential stratification prior to randomization. Criteria for stratification will be malaria prevalence levels and/or demographic endpoints. Following stratification (as needed), clusters will be allocated to receive the SR product either through delivery by study personnel (reference arm, positive control), VHTs, or vouchers using a random number generator (https://www.random.org). The cluster allocation code will be made available to the DSMB for use in safety assessments. The site database manager will assign a unique HH identification number (HIN) to each HH for use in monitoring malaria infections during the trial and the site intervention administrator will coordinate SR product distribution to mitigate stock-out. Following cluster allocation, a total of 45 households will be randomly selected per cluster. In HHs with multiple children that meet the study inclusion criteria, all eligible children will be invited to participate; however, only one will be enrolled. The methods used for this randomization will be the rand() function from Excel for households and the random.sample() function for age-eligible members.

### Concealment mechanism {16b}

The Sponsor, study Principal Investigator (PI), and staff will not be blinded to cluster assignment (SR delivery channel). There will be no placebo integrated into the study design, as the SR intervention will have already been evaluated for efficacy under controlled conditions. Ethical approvals and census and mapping activities were conducted prior to the allocation. However, cluster allocation was shared with the study team prior to recruitment and enrollment to allow the identification of distribution points within clusters in the voucher arm and the recruitment of VHTs within the VHT arm.

### Implementation {16c}

Trained study staff will be responsible for the management of product implementation which will include the initial deployment of product, subsequent removal, and replacement at 4-week intervals.

## Assignment of interventions: blinding

### Who will be blinded {17a}

The Sponsor, study PI, and staff will not be blinded to cluster assignment (SR delivery channel). There will be no placebo integrated into the study design, as the SR intervention will have already been evaluated for efficacy under controlled conditions.

### Procedure for unblinding if needed {17b}

Not applicable—study will not be blinded.

## Data collection and management

### Plans for assessment and collection of outcomes {18a}

#### Mapping of the study area and census measurements

Mapping will be conducted in all 48 clusters; 70% refugee and 30% host villages will be considered. These will be selected based on government structures and population size of the clusters and proximity to the refugee villages. All HHs in each cluster will be invited to participate in the study. Verbal consent will be obtained and within each cluster, individual HHs will be enumerated and all structures in the study area will be mapped using GPS coordinates and assigned a unique HIN. A questionnaire will be administered to measure housing structure and HH member demographic characteristics. This includes housing construction, and number of inhabitants and their age.

Following mapping, clusters will be delineated and randomly allocated to delivery channels using standard statistical software (e.g., SAS, STATA, or R).

#### Enrollment of the cohort

A cohort of children aged ≥ 6 months to ≤ 59 months will be enrolled within each cluster to assess the effectiveness of the SR product against the contemporaneous control and in additional clusters to estimate effectiveness using programmatic delivery systems. HHs will be randomly selected from the master list of HHs obtained during ramp-up phase mapping to achieve the desired sample size.

Selected HHs based on cluster maps will be visited and if a child is present, the study will be explained and consent obtained from the child’s primary caregiver before screening for eligibility and enrolling him/her in the study. All eligible children in a selected HH will be invited to participate. (See above for a more complete description of the consent process.) At enrollment, the study team will have a list of all age-eligible children in each HH selected. These data will have come from the initial mapping and census of the study clusters. At the time of participant recruitment, the tablet will randomly select one of the age-eligible children for the field staff to screen for inclusion and exclusion criteria, which includes a hemoglobin test. Prior to screening, the team will obtain parental/caregiver consent. If there are no eligible children in the HH after screening, a new HH will be randomly selected from the HH listing exercise (mapping and census).

At enrollment, the age and gender of the child will be recorded and the parent/guardian (as needed) will be asked about the use of ITNs and anti-malarial drugs. Contact information, as available, will be captured. A fingerstick blood sample will be taken for a malaria RDT, and a hemoglobin level will be evaluated in a capillary sample using the HemoCue device**.** The study team will work with local health partners and the district health department providing iCCM to make sure children in need of iron supplements and antihelminths are managed according to national protocols and coordinate any referrals or follow-ups as needed. If the screened child does not meet the inclusion criteria, the tablet will randomly select another child from the remaining age-eligible children in the same house. The process will continue until one of the children is successfully enrolled.

All children enrolled in the study will be provided an ITN. This will allow us to measure the added benefit of SR product above that provided by currently recommended preventive measures. In addition, a blood sample will be taken for a RDT for all participants enrolled in the cohort to be used as a baseline data measure. All participants (regardless of RDT results) will be provided a treatment dose of artemisinin-combination treatment (ACT) AL at the start of the study prior to receiving SR, to clear any prepatent or patent malaria parasites, unless they have recently been treated (within the last 2 weeks). Participants will be treated according to RDT results throughout the study period, according to national protocols.

The enrollment period will take approximately 1 month, during which time incidence of malaria disease in study clusters will be measured prior to the deployment of the study product.

#### Enrollment to receive the SR product

The study product will be explained to the head of HH, as described in the main protocol, and consent for participation will be obtained prior to start of study. No routine blood samples will be sought.

#### Follow-up of the cohort

All efforts to overlay the commencement of enrollment with the start of the malaria transmission season will occur; however, programmatic timelines may not allow for perfect alignment. The 12-month follow-up period will ensure estimating potential changes in malaria incidence during anticipated seasonal malaria transmission peaks.

The distribution of the SR will occur after enrollment. At this time, cohort participants will be presumptively cleared of parasites with a treatment dose of AL unless they have recently been treated (within the last 2 weeks). This will be done so that the incidence of malaria infection following study product deployment can be measured.

Participants enrolled in the cohort will be followed every 2 weeks for malaria infection during the 12-month period after the study product has been distributed. All cohort participants and HH residents will also be informed to come to the nearest health facility for unscheduled sick visits, as needed. If the participant presents with a history of fever in the last 48 h, a RDT will be performed and AL will be administered if the RDT is positive for malaria.

Every 2 weeks, study team data collectors will visit the enrolled HHs. At each data collection HH visit, the parent/guardian will be asked about the recent use of ITNs and other vector control interventions as well as recent history of illness, recent travels outside the HH, and recent use of anti-malarial drugs.

During the first scheduled HH visit, a blood sample will be taken for malaria RDT (Monthly Visit #1). During the second scheduled HH visit, a blood sample will only be taken if the participant has a recent history of fever (Monthly Visit #2). The total blood volume for samples at each visit will not exceed 500 mli. A second blood sample may be taken for hemoglobin testing during follow-up only if the data collection/clinical team suspects anemia in participating children.

Cohort participants who test positive for malaria by RDT will be treated with ACTs free of charge according to national treatment guidelines. Hospital fees will not be paid by the study unless the illness or injury is due to study products or procedures as determined by the health facility physician and the study coordinator, in coordination with the study’s local PI. If there is illness or injury due to study products or procedures, fees will be paid for care at the government health facilities or District or Provincial Hospital according to the clinical trial insurance policy of the study.

When participants are not available for a follow-up visit, there will be a grace period/ “window” for each follow-up visit. There will be an allowable range of + / − 3 days for scheduled follow-up data collection home visits to occur.

In total, it is estimated that approximately 2.5 h will be needed from research participants at the start of the study (for the HH mapping, structures and HH enrollment, parasite clearance, and distribution of ITN). Throughout the study, 50 min will be needed for the VHT and study personnel arms (for the follow-up visits and SR placement), while approximately 1 h may be needed for the voucher arm as they will be walking to the central location to redeem their SR.

#### Cost-effectiveness

We will address issues of cost of implementation by evaluating efficacy in relation to coverage using varied delivery systems. The cost of manufacturing the SR intervention by SCJ will be monitored and documented to model affordable pricing by industry stakeholders to produce next in class SR products ensuring efficacious options for procurement by donor agencies and countries. Projected cost per person protected will comprise product costs based on models using project data (epidemiological, and market) and operational costs. Treatment cost averted using ITNs vs ITNs + SR to the global scale is required for estimating the economic impact. The study will measure the cost of Mosquito Shield implementation in relation to manufacturing, efficacy, and coverage to model projections of cost-effectiveness to incentivize procurers.

### Plans to promote participant retention and complete follow-up {18b}

Participant retention strategies will include participant tracing when they miss appointments, visit reminders, request participants to inform study staff of moves outside or within the study area, foster relationships with participants, provision of study staff contact information for easy communication to alleviate any concerns, and periodic generation of retention rate to evaluate strategies. Utilization of the community structure (e.g., VHTs) will make retention efforts more efficient and effective. A study SOP will be developed to provide more details on retention activities to be conducted by study staff.

### Data management {19}

A combination of standardized paper-based and digital forms (under Android tablets) will be used in Uganda. CRS and UND/Center for Research Computing (CRC) will work together to develop the quantitative forms to be uploaded on Android phones/tablets. All data issuing from the electronic data collection system will follow the same data collection processes outlined in the paragraph below. Any changes to quantitative data forms will need to be agreed by relevant Institutional Review Boards (IRBs), the overall CRS PI (in consultation with CRS’ technical advisors), Infectious Diseases Research Collaboration (IDRC)’s local PI, the CRS country level study Program Manager, and UND’s PI. In the event that all four cannot find a consensus on proposed changes, UND’s PI will make the final decision.

#### Data storage

Any data collected on paper forms (including consent forms) will be scanned and transferred to binders for storage in a secure and locked restricted access area, while all electronic captured data will be archived with a documented history of changes or corrections at the local study site. Using CommCare, CRS and IDRC will collect data which will be securely stored on Android devices and then synchronized to CommCare cloud. Data will then undergo an initial cleaning and de-identification by CRS/IDRC, before being synced to UND’s central database. Data from CRS to UND will be transferred through a dedicated secure sFTP server with password protect access from CRS.

#### Data quality control and quality assurance

Data management staff members will have the responsibility of data verification for accuracy and assuring data collection follows standardized protocols. These activities will promote high quality of data and ensure the trial is performed in compliance with GCP and the applicable regulatory requirement(s). Training on data collection will occur prior to the start of intervention and throughout the trial period with refresher trainings. Standardized data collection forms will be used and source data verification will occur through (1) self-quality checks, making sure data forms are fully completed and (2) data queries, quality checks on a routine basis. In addition, tablet-based digital forms that will be used for data entry will be custom-designed to include rules and conditions for data variable responses (e.g., text responses cannot occur for numeric value, and thresholds for numeric data, etc.).

#### Data sharing

This project will generate considerable data over the course of the 1-year study period. The data management plan will follow the guidelines and suggestions put forward by the NIH in its online guidance document https://humansubjects.nih.gov/data_safety, the WHO Data Sharing Policy https://www.who.int/publications/i/item/9789240044968, and by the respective institutions involved in the research (CRS, IDRC, and UND). The goal is transparent sharing of key findings and data so that the broad impacts of the research are meaningful and useful to key stakeholders and will therefore be shared with stakeholders as may be required.

### Confidentiality {27}

A password-protected central study database warehousing data will be developed and managed by the UND and serve as a data repository and utilized for safe data storage, extraction, integration, and analysis. The data warehouse and file repository will be backed up weekly at the local server level to ease recovery as needed. In addition, data is stored and backed-up on CommCare cloud. Access to study data is controlled through centralized administration and access will be granted to all study investigators through UND PI’s permission. Research records for all study participants including history and physical findings, and results of consultations are to be maintained by the local site PI in a secure storage facility and by the UND and CRS, for a minimum of 5 years after the end of the project or until notified by grantee, and for a maximum of 7 years. Data and samples can be destroyed at any given time after those 5 years.

All records will be kept confidential. Study participants will be identified only by a unique identification number. No individual identities will be used in any reports or publications resulting from the study. All project staff will be trained on procedures for maintaining confidentiality.

### Plans for collection, laboratory evaluation, and storage of biological specimens for genetic or molecular analysis in this trial/future use {33}

There is no anticipated future use of participant blood samples. These samples will be taken only for purposes of RDT diagnosis of malaria infection in the current study. The Study Sponsor, UND, will be responsible for managing requests for access to study data in partnership with CRS according to the data management policies of the funder.

## Statistical methods

### Statistical methods for primary and secondary outcomes {20a}

The intention to treat (ITT) analysis is the primary analysis approach for both the primary objectives. The ITT population includes the first recruited participant from each recruited HH that receives at least one SR product per the cluster randomization schedule and is analyzed according to the randomization schedule.

The primary objective will be examined by comparing the HRs of the first-time malaria infection between each of the two real-life product delivery mechanisms vs. the reference mechanism in the ITT population. The complementary log–log (cloglog) regression model log(-log(1- Θ_kjit_)) = β_0t_ + **x**^t^_kji_**β**_1_ + z_k_ + z_j(k)_ will be applied. Θ_kjit_ is the discrete time HR of participant i from HH j in cluster k at time t, and x_kji_ contains the individual-, HH- and cluster-level factors that include the treatment group (reference, VHT, voucher) and other relevant covariates (e.g., age, gender, HH type, cluster size, etc.). If the data are extremely unbalanced in a categorical covariate (e.g., 99% HHs had the same type of walls) or if a non-ignorable portion of the participants have missing values on a covariate (due to missing at random (MAR) or missing completely at random(MCAR), that covariate may be excluded in the model. z_k_ ~ N(0,σ_1_^2^) and z_j(k)_ ~ N(0,σ_2_^2^) are the random effects at the cluster and HH levels respectively (z_j(k)_ will be only necessary if some HHs contribute more than one individual to the study).

The difference in effectiveness between each of the two real-life delivery mechanisms vs the reference mechanism will be estimated by exp ($\widehat{\beta }$), where $\widehat{\beta }$ is the estimated regression coefficient associated with the real-life delivery mechanism compared to the reference. The Wald-type 90% confidence interval will be obtained for the HR along with the point estimate exp($\widehat{\beta }$). The cloglog model used to analyze the overall infections is similar to the above model with one modification; that is, the cloglog model will include an additional random effect z_i(jk)_ ~ N(0,σ_3_^2^) to capture the correlation among the overall events from the same participant.

The frequency and percentage of AEs and SAEs across clusters among enrolled participants will be summarized by the treatment arm. The AE/SAEs summary will be provided for both clinical diagnoses as well as symptoms. In addition, they will be labeled by Probable, Possible, Plausible (such as dermal events, oral events, Inhalation events), or Unlikely (eye Irritation, headache) due to SR.

Through carrying out a costing study, modeling activities planned in the Project will facilitate projections of SR cost-effectiveness of delivery at scale (using VHTs and vouchers) based on efficacy in specific contexts and delivered as either a stand-alone tool or when combined with other disease control strategies at scale. By identifying effective delivery systems, the operational costs will be reduced making SRs more affordable for MOHs and more feasible for development partners.

### Interim analyses {21b}

No interim analysis will be performed on the epidemiological data collected from the intervention period post-randomization.

### Methods for additional analyses (e.g. subgroup analyses) {20b}

The per-protocol (PP) population includes the participants from the ITT population who are treated following the specifications of the study protocol without major protocol deviations.

#### Participants who move to a new house during the intervention follow-up period

- For a participant who moves to a different house within the same cluster, that participant will be included in both the ITT and PP analyses. The HH characteristics will be updated at the time the subjects moved.
- For a participant who moves to a different house in a different cluster, the data from the participant before the participant moves will be included in the ITT analysis. All data from the participant will be included in the PP analysis, both the treatment information and the HH characteristics will be updated at the time the participants moved.

#### Participants who are hospitalized for serious complicated illness (e.g., chronic illness), die, drop out, or miss scheduled visits due to reasons not related to the malaria outcome or intervention during the follow-up period

For participants that fall under this category, the available data from the participants (up to the time point when the participants are hospitalized, die, or drop out; data from the scheduled visits that the participants did not miss) will be included in both the ITT and PP analyses as the missing or absent data can be ignored.

#### Participants who do not receive (complete) intervention due to traveling outside, mis-application, or partial application of the product

For the ITT analyses, these participants will be included as is. For the PP analysis, “travel outside” (Y or N; an individual-level covariate) and the product application rate in each HH (expected to be close to 100%) will be included as covariates if the data are not overly imbalanced between the Y and N categories for “travel outside”, and there is practically/clinically.

The primary analyses will also be carried out in the PP population, with some modification on the covariate list in the cloglog models for the first-time infection and the overall infection. Specifically, for the PP analysis, “travel outside” (Y or N; an individual-level covariate), and the product application rate in each HH (expected to be close to 100%), the coverage rate of the product in each cluster, will be included as covariates if the data are balanced between the Y and N categories for “travel outside” and there is practically/clinically meaningful variation in the product application rate across HHs and clusters and in the coverage rate across clusters.

For both the ITT and PP analysis and in the cloglog models for the first-time infection and the overall infection, in addition to the HH-level covariates listed above (number of doors, open eaves or not, and wall type), we will also perform a supplementary analysis by including bednet usage in the last 24 h as an additional covariate, which is a post-randomization covariate.

The first and overall malaria incidence rate per person-year during the follow-up will be calculated for each treatment arm. Since the malaria incidences are interval-censored, the mid-point between two visits will be imputed as the time at risk for a malaria event. The average incidence rate within each arm will be calculated, together with the coefficients of variation.

### Methods in analysis to handle protocol non-adherence and any statistical methods to handle missing data {20c}

Standard operating procedures have been developed for all study activities and a Clinical Monitoring Plan has been established among UND, CRS, and IDRC. Departures from the protocol that are not participant-specific will be documented in a Protocol Deviation report developed by CRS and reported as required, and the site re-educated as necessary. Any participant-specific non-compliances and other protocol deviations will be captured in the protocol deviation Case Report Form developed by CRS and filed as hard copies. Major protocol deviations will be submitted via email to applicable IRBs and the Sponsor within 24 h of PI becoming aware of them, and followed by a detailed report, within 7 working days.

Significant effort will be made to avoid missing values on the outcome (malaria infection status and visit dates, entomological endpoints). When missing values occur for an outcome for reasons not related to the outcome, reasons for missingness and the missing fraction by treatment arm and cluster will be reported. Per protocol, the participants are screened actively on their malaria status (the outcome) every 4 weeks.

If a participant misses one or more scheduled visits due to reasons not related to SRs or the outcome, the participant will have missing values on the outcome that can be regarded as ignorable missingness (missing at random (MAR) or missing completely at random (MCAR)). If a participant drops out study due to reasons unrelated to SRs and/or malaria infection, then the missing observations from the participant can be regarded as ignorable missingness (MAR or MCAR). In both cases, all available data from the participant will be included in the primary and secondary analysis, without employing any specific technique to deal with the data.

Missing baseline covariates (individual-level, HH-level, cluster-level) that are a part of the regression models for the outcome of interest will be imputed using simple hot-deck imputation methods if the missing fraction for the covariate is < 5%. If the missing fraction for a covariable are ≥ 5%, appropriate multiple imputation approaches will be applied. If a non-ignorable portion of the participants will have missing values on a covariate (due to missing at random or missing completely at random), that covariate may be excluded in the model.

### Plans to give access to the full protocol, participant level-data and statistical code {31c}

The Statistical Analysis Plan (SAP) and analytic code will be made open access. Data and supporting information will be made available 12 months following the completion of data analysis and will remain open access in the public domain.

## Oversight and monitoring

### Composition of the coordinating center and trial steering committee {5d}

UND will serve as the lead organization for this program and will assume the overall responsibility for management, oversight, and administration of the program. The coordinating personnel at UND will include the Lead PI, Scientific Director, Program Manager, and Finance Manager. UND will communicate on a day-to-day basis with CRS and IDRC. CRS and IDRC will be responsible for running the study on a day-to-day basis which includes, but will not be limited to deploying SRs and participant follow-up. Representatives from CRS, IDRC, and UND will all serve on the data management team to oversee the development and implementation of data collection, recording, and cleaning.

### Composition of the data monitoring committee, its role and reporting structure {21a}

The DSMB reviews safety data about the cRCT on an ongoing basis in order to monitor and rapidly identify any accumulating safety issues from across the program and may provide recommendations about stopping the study for safety reasons. Additionally, the DSMB provides additional credibility to study quality, by reviewing summary reports from PIs during baseline and intervention phases and making recommendations as needed about adjustments for study quality reasons. The DSMB consists of a Chair, Medical Monitor, DSMB statistician, and independent statistician.

Safety data should be reviewed routinely and regularly by the DSMB Medical Monitor. If significant concern would be raised, he/she can engage with the committee. Summary of AEs, SAEs, and death reports observed during the studies should be reviewed by the entire committee at pre-determined checks (quarterly). This should include a comparison of the rate of AE and SAE in the three study arms and at the individual study site. The DSMB will be notified of any SAEs that are “at least possibly related” to the research study as they are reported to PIs. Unblinded efficacy data will be analyzed according to a pre-defined SAP by an Independent Statistician when pre-defined enrolment targets have been achieved. The role of the DSMB statistician will be to agree to what information will be reviewed (see below) and then review and interpret this information with the other DSMB members, perhaps to request further analysis, for example. The DSMB statistician will contribute input to program PIs as to what subset of the SAP is to be presented at different meetings.

Members generally have no ongoing financial relationship with a trial's commercial sponsor and will not be involved in the trial conduct in any role other than that of a DSMB member. Prospective members will be asked to disclose their financial relationships with any of the sponsors and/or their competitors. The DSMB reports to the Sponsor, UND. The DSMB charter can be made available upon request to UND.

### Adverse event reporting and harms {22}

An AE includes any noxious, pathological, or unintended change in anatomical, physiological, or metabolic functions as indicated by physical signs, symptoms, and/or laboratory-detected changes occurring in any phase of the clinical study associated with the study intervention. This definition includes an exacerbation of pre-existing conditions or events, intercurrent illnesses. A SAE is any untoward medical occurrence that results in death, is life-threatening, results in persistent or significant disability/incapacity, requires in-patient hospitalization or prolongation of existing hospitalization, or is a congenital anomaly/birth defect in the offspring of a study participant. In addition, important medical events that may jeopardize the participant or may require intervention to prevent one of the other outcomes listed above will be considered serious.

All SAEs will be recorded on the appropriate SAE case report form, followed through resolution by a study physician, and reviewed by a study physician.

Symptomatic uncomplicated or severe malaria infection will be coded as AEs as will any other acute illness, throughout the 12 months of the study follow-up phase. Clinical malaria will be recorded in the same fashion as other AEs. In general, uncomplicated malaria will constitute an AE and severe malaria will constitute an SAE. Anticipated day-to-day fluctuations of pre-existing conditions that do not represent a clinically significant exacerbation need not be considered AEs. Discrete episodes of chronic conditions occurring during a study period will be reported as AEs to assess changes in frequency or severity. Pre-existing conditions or signs and/or symptoms (including any that are not recognized at study entry but are recognized during the study period) present in a participant prior to the start of the study will be recorded on the participant’s Case Report Form.

AEs will be documented in terms of a medical diagnosis. When it is not possible to make a specific medical diagnosis, the AE will be documented in terms of signs and/or symptoms observed by the investigator or reported by the participant at each study visit. Any hospitalization will be considered a SAE.

AEs to be recorded as endpoints will be pre-defined based on SCJ toxicology reports of “probable,” “possible,” “plausible,” and “unlikely”:

- Probable: sensory irritation (oral and dermal)
- Possible: nausea/vomiting (oral), skin irritation/rash (dermal), runny nose (inhalation)
- Plausible: salivation
- Unlikely: eye irritation, headache

AEs and SAEs will be recorded throughout the study. SAEs and the study reports will also be provided to the Makerere University School of Public Health Research and Ethics Committee as required by their current Standard Operating Procedures. AEs will be recorded at time of product replacement and/or when a participant reports for malaria testing. In addition, participants will be informed they can contact study staff anytime they experience an AE. SAEs will be recorded at time of death, hospitalization. Assessment of SAE relatedness to intervention will be conducted by the health facility physician and the study coordinator, in coordination with the local PI in-country with confirmation/verification by the DSMB Medical Monitor based on the study clinician SAE report and trends in other SAE datasets.

### Frequency and plans for auditing trial conduct {23}

Trial conduct will be monitored through direct observation throughout the study period. Audit frequency will be directly related to timing of study procedures related to participant recruitment, enrollment, screening, and follow-up. Auditors will consist of study supervisors with expertise and experience with the specific procedure.

### Plans for communicating important protocol amendments to relevant parties (e.g. trial participants, ethical committees) {25}

Protocol amendments will be submitted to the Sponsor and local IRBs as well as the WHO Ethical Review Committee (ERC) for approval. Any amendments, outcomes, analyses, or more, will be communicated in person and virtually. Face-to-Face meetings will be held between the Lead PI, scientific director, site staff, and members of the site-specific countries where the trials are being conducted. These meetings will include National Malaria Control Programs, MOHs, other in-country public health officials, members of civil society, religious leaders, and key beneficiaries. These meetings will be critical as the trials will be in-progress and the topics addressed will be pertinent to facilitating further execution of the trial. Due to the project design and geographic location of the study team, teleconferences will be another method of formal project communication. Stakeholder teleconferences will be scheduled as needed to review and assess study progress and issues. Agendas for all teleconferences will be drafted by UND and distributed.

### Dissemination plans {31a}

Dissemination of results includes submission to WHO, workshop with study partners, on-site meetings in Uganda, and presentations at scientific meetings and/or peer-reviewed publications.

Per journal requirements, we anticipate sharing analytical datasets used to generate the outcomes presented in publications. These de-identified datasets may be used by other researchers who want to independently repeat analyses.

## Discussion

SR products are envisioned to complement existing vector control methods through the continual release of volatile AIs. Recent outcomes from clinical trials in Kenya [16, 17], and Peru [18], have indicated SR products to protect against malaria and dengue human infection, respectively. However, evidence gaps remain to support full assessment by the WHO Vector Control Advisory Group regarding the public health value of a SR product category [19]. Data from two ongoing clinical trials are meant to fill this gap [19, 20]. While the efficacy of a SR to reduce pathogen infection within a large-scale may be demonstrated through a series of clinical trials, it is unlikely that efficacy estimates derived from tightly controlled phase III trials will be realized in program settings. The current Operational Research study in Uganda has been designed to complement these trials by generating findings on optimal delivery processes for SR products, estimating SR cost-effectiveness in a 'real-world setting, and assessing user perceptions of the SR intervention in sub-Saharan Africa where underlying transmission rates are historically high and traditional mosquito control measures are often not available or easy to implement. Outputs are meant to specifically inform policy makers on whether to recommend SRs as a means to further reduce malaria transmission for such operational programs serving refugees and those who are forcibly displaced, as well as, support a global policy endorsement for the use of SRs more broadly by guiding the scientific, regulatory and social parameters that constitute an outline for optimum product characteristics.

## Trial status

Under protocol version 12 from October 12, 2023, recruitment, screening, and enrolment of participants for follow-up with intervention is anticipated to commence in July 2024 and be completed within 1 month (August 2024).

## Data Availability

The SAP and analytic code will be made open access. The data and supporting information will be made available 12 months following completion of data analysis and will remain open access in the public domain. Open-access repository distributed under the terms of the Creative Commons Attribution (CC-BY) License, which permits unrestricted use, distribution, and reproduction in any medium, provided the original author and source are credited.

## Abbreviations

ACT: Artemisinin-combination treatment
AE: Adverse event
AEGIS: Advancing Evidence for Global Implementation of Spatial Repellents
AI: Active ingredient
AL: Artemether lumefantrine
CRC: Center for Research Computing
cRCT: Cluster randomized control trial
CRS: Catholic Relief Services
DSMB: Data Safety Monitoring Board
ERC: Ethical Review Committee
HH: Household
HIN: Household Identification Number
HR: Hazard rate
iCCM: Integrated Community Case Management
ICF: Informed consent form
IDRC: Infectious Diseases Research Collaboration
IRB: Institutional Review Board
IRS: Indoor residual spraying
ITN: Insecticide-treated net
ITT: Intention to treat
LAR: Legally Authorized Representative
MAR: Missing at random
MCAR: Missing completely at random
MOH: Ministry of Health
PI: Principal Investigator
PP: Per-protocol
RDT: Rapid diagnostic test
SAE: Serious adverse event
SAP: Statistical Analysis Plan
SCJ: SC Johnson, A Family Company, Inc.
SR: Spatial repellent
UND: University of Notre Dame
VHT: Village Health Team
WHO: World Health Organization
